# Cognitive Performance at Time of AD Diagnosis: A Clinically Augmented Register-Based Study

**DOI:** 10.3389/fpsyg.2022.901945

**Published:** 2022-07-01

**Authors:** Minna Alenius, Laura Hokkanen, Sanna Koskinen, Ilona Hallikainen, Tuomo Hänninen, Mira Karrasch, Minna M. Raivio, Marja-Liisa Laakkonen, Johanna Krüger, Noora-Maria Suhonen, Miia Kivipelto, Tiia Ngandu

**Affiliations:** ^1^Department of Psychology and Logopedics, University of Helsinki, Helsinki, Finland; ^2^Population Health Unit, Finnish Institute for Health and Welfare, Helsinki, Finland; ^3^Unit of Neurology, Institute of Clinical Medicine, University of Eastern Finland, Kuopio, Finland; ^4^Neurology of Neuro Center, Kuopio University Hospital, Kuopio, Finland; ^5^Department of Psychology, Abo Akademi University, Turku, Finland; ^6^Department of General Practice, University of Helsinki, Helsinki, Finland; ^7^Unit of Primary Health Care, Helsinki University Hospital, Helsinki, Finland; ^8^Geriatric Clinic, Department of Social Services and Health Care, Laakso Hospital, Helsinki, Finland; ^9^Research Unit of Clinical Neuroscience, Neurology, University of Oulu, Oulu, Finland; ^10^MRC, Oulu University Hospital, Oulu, Finland; ^11^Department of Neurology, Oulu University Hospital, Oulu, Finland; ^12^Division of Clinical Geriatrics, Center for Alzheimer Research, NVS, Karolinska Institutet, Stockholm, Sweden; ^13^Institute of Public Health and Clinical Nutrition, University of Eastern Finland, Kuopio, Finland; ^14^Ageing Epidemiology Research Unit, School of Public Health, Imperial College London, London, United Kingdom

**Keywords:** cognitive screening, cognitive performance, Alzheimer’s disease, CERAD, timely diagnosis, mixed dementia, register-based study

## Abstract

We aimed to evaluate the feasibility of using real-world register data for identifying persons with mild Alzheimer’s disease (AD) and to describe their cognitive performance at the time of diagnosis. Patients diagnosed with AD during 2010–2013 (aged 60–81 years) were identified from the Finnish national health registers and enlarged with a smaller private sector sample (total *n* = 1,268). Patients with other disorders impacting cognition were excluded. Detailed clinical and cognitive screening data (the Consortium to Establish a Registry for Alzheimer’s Disease neuropsychological battery [CERAD-nb]) were obtained from local health records. Adequate cognitive data were available for 389 patients with mild AD (31%) of the entire AD group. The main reasons for not including patients in analyses of cognitive performance were AD diagnosis at a moderate/severe stage (*n* = 266, 21%), AD diagnosis given before full register coverage (*n* = 152, 12%), and missing CERAD-nb data (*n* = 139, 11%). The cognitive performance of persons with late-onset AD (*n* = 284), mixed cerebrovascular disease and AD (*n* = 51), and other AD subtypes (*n* = 54) was compared with that of a non-demented sample (*n* = 1980) from the general population. Compared with the other AD groups, patients with late-onset AD performed the worst in word list recognition, while patients with mixed cerebrovascular disease and AD performed the worst in constructional praxis and clock drawing tests. A combination of national registers and local health records can be used to collect data relevant for cognitive screening; today, the process is laborious, but it could be improved in the future with refined search algorithms and electronic data.

## Introduction

For people aged 60 years and over, the prevalence of dementia increases exponentially with age (Prince et al., 2015). Alzheimer’s disease (AD) is the major cause of dementia (Prince et al., 2015). The neuropathological process of AD occurs over a period of years, and the diagnosis can be made at the following different stages (Prince et al., 2011): earliest possible diagnosis in the event that new reliably predictive biomarkers are developed (T1); earliest possible diagnosis using currently available technology (T2); “timely” diagnosis, responding to patient and caregiver concerns rather than proactively screening for the disease (T3); and “late-stage” diagnosis (T4). Within past years the currently available technology T2 has evolved for example from traditional use of magnetic resonance imaging (MRI) and computed tomography CT (Frisoni et al., 2010) to artificial intelligence enabled brain imagining analyses (Frizzell et al., 2022) like CT-automated analysis method when MRI is inaccessible or contraindicated (Kaipainen et al., 2021). Also on-going is for example AD biomarker discovery and development from cerebrospinal fluid (CSF) to the next generation of biomarkers (Carvalho et al., 2022). T3 states can be further divided into initial detection of cognitive difficulties (T3.1); assessment with specialist referrals to decide whether symptoms are due to the dementia syndrome (T3.2) and if dementia is present, to achieve a diagnosis of cause/subtype, staging, and relevant comorbidities (T3.3); and care planning to address current and future needs of the patient and family (T3.4; Brooker et al., 2014). Timely diagnosis T3 process may be challenged with different level of subjective cognitive impairment (Sutherland et al., 2022) or lack of self-awareness about having a disability (Cacciamani et al., 2021). If the information given by patient or caregiver is inaccurate, the diagnosis may be delayed. Late-stage diagnosis T4 is then not made at all or made very late in the process by which time cognitive impairment, disability and behavioral symptoms are all quite marked (Prince et al., 2011).

A recent international study (Podhorna et al., 2020) reported that approximately half of the patients received a subtype-level diagnosis (T3.3) within 6 months after presenting with initial symptoms (T3.1). However, there were large national differences, ranging from 35% in France to 65% in Japan. Many caregivers (47%) experience that the diagnosis of dementia is set too late (Woods et al., 2019). The most frequent contributing factors for late diagnoses were that the patient with dementia refused to be assessed (38%), the first professional seen did not consider anything was wrong (33%), and caregivers/patients assuming the problems were “just old age” (26%).

An increasing number of studies are based on data from very large registers. In the AD field, register-based data have been used, e.g., to investigate incidence and prevalence of AD/dementia (Tolppanen et al., 2016; Kivimäki et al., 2018; Zakarias et al., 2019; Ponjoan et al., 2020), and AD classification (Ford et al., 2020). Registry-based data have also been used to evaluate medication use, healthcare service use (Tolppanen et al., 2016), and quality of diagnostic processes (Zakarias et al., 2019). Often, when register-based data are used, it is assumed that diagnoses in the register as well as the utilized criteria and decision-making logic are timely and accurate across different geographical regions and healthcare service providers. Few studies have assessed whether this is actually the case. To our knowledge, based on real-world healthcare register data no previous study has assessed AD stage and cognitive performance of persons at the time of AD diagnosis.

With the proportion of the population aged 65 years and over growing rapidly and the proportion of those aged 80 years and over growing even faster [Official Statistics of Finland (OSF), 2019], more up-to-date methodologies are needed for larger scale cognitive screening. Despite the proposals to move toward purely biological definition of AD within research framework (Jack et al., 2018), AD diagnosis is in most settings still made through identification of typical clinical features (McKhann et al., 1984, 2011; American Psychiatric Association, 1994; Association, 2013). The Consortium to Establish a Registry for Alzheimer’s Disease (CERAD) neuropsychological battery (CERAD-nb) was translated and introduced in Finland more than 20 years ago (Hänninen et al., 1999). During years 2006–2008 national guidelines for early diagnosis, treatment, and care management of dementia were given (Hänninen et al., 2010; Sotaniemi et al., 2012; Working group set up by the Finnish Medical Society Duodecim, 2016). CERAD-nb replaced use of MMSE only and was transformed from an assessment tool to a cognitive screening tool used mainly in primary care by nurses and occasionally by physicians in primary care or in private sector (Suhonen et al., 2008; Working group set up by the Finnish Medical Society Duodecim, 2016). In the guidelines (Working group set up by the Finnish Medical Society Duodecim, 2016), a comprehensive assessment by neuropsychologist in specialized unit is recommended mainly for more complex cases such as younger (under 65 years) or highly educated or clinically atypical patients. Originally, the CERAD-nb was introduced as a measure for early cognitive impairment in AD (Morris et al., 1989). Compared with late-onset AD (LOAD), early-onset AD (EOAD; Migliaccio et al., 2012; Palasí et al., 2015) or mixed cerebrovascular disease and AD pathologies (AD_CVD; Eckerström et al., 2020) more often present earlier with symptoms other than the progressive memory deficit typical for LOAD.

National registers are unique resources since they provide nationwide coverage of real-world data on AD diagnosis. Complementing the register data with cognitive screening data and clinical information from local healthcare records enables investigation of the timing of AD diagnosis and cognitive performance in the CERAD-nb at the time of diagnosis. In a population identified through healthcare registers at the time of AD diagnosis, the main research questions are as follows:

1. What is the overall disease stage and what is the proportion of AD patients who have completed CERAD-nb at a mild stage of the disease?
2. What is the distribution of AD subtypes? What characterizes their cognitive performance profile?
3. What can be learned from the data collection process?

## Materials and Methods

During the time of the study, Finland’s healthcare comprised municipal, occupational, and private facilities. Municipalities fund and organize the provision of primary care and form 20 secondary care hospital districts to fund and provide hospital care (OECD, 2017). Private facilities include both outpatient and inpatient care services.

### Study Population

Patients with their first recorded AD diagnosis were identified from two Finnish national health registers (NHR), which consist of primary and secondary care treatment notifications: Register of Primary Health Care Visits Avohilmo (Finnish Institute for Health and Welfare Register of primary health care visits, 2021b) and Care Register for Health Care Hilmo (Finnish Institute for Health and Welfare Care register for health care, 2021a). Diagnoses have been coded according to the International Statistical Classification of Diseases and Related Health Problems, 10th revision (ICD-10) or the International Classification of Primary Care (ICPC-2). The initial sampling was based on ICD codes F00 and G30 and their subcodes, and the ICPC-2 code P70. The target was to identify all patients aged 60–80 years at the time of AD diagnosis in five geographical areas (in and around the cities of Helsinki, Kuopio, Turku, Oulu, and Seinäjoki). Patients were excluded if before AD diagnosis they had dementias other than AD, other major neurological or psychiatric or developmental disorders impacting cognition, or during the previous 24 months a diagnosis of serious mood disorders or alcoholism or other transitory psychiatric/neurological disorders affecting cognition. In terms of age, gender, and geographical location, the sampling focused on patients comparable to the participants of the Finnish Geriatric Intervention Study to Prevent Cognitive Impairment and Disability (FINGER; Ngandu et al., 2014), which formed the non-demented comparison group (controls). For the identified individuals, clinical and cognitive information was requested from local health records (LHRs). This study was approved by the Finnish Institute for Health and Welfare, decision THL/1649/6.02.00/2018; additionally, local approvals from service providers were obtained as needed.

First, a pilot sampling from the NHR registers targeted the AD diagnosis given during 2009–2011. This was chosen to match the control data collection. The information received from the LHRs for the first approximately 100 patients revealed that selected search criteria were unfeasible; the primary care interfaces from the LHRs to the NHR were gradually built since 2008, and therefore, what appeared as a new diagnosis in NHR was in fact a diagnosis related to a follow-up visit. This created a need to modify the sampling. A second sampling from NHR registers targeted AD diagnosis during 2010–2013. LHR data were collected from seven public service providers: primary healthcare services of Helsinki (*n* = 486), Turku (*n* = 229), Kuopio (*n* = 129), and Oulu (*n* = 44); university hospitals of Kuopio (*n* = 76) and Oulu (*n* = 29); and the central hospital of Seinäjoki (*n* = 79). Three major medical units and several smaller ones were excluded: one because of the requested costs (*n* = 132), 2 units (*n* = 90, *n* = 29) because of the impact of COVID-19 on data collection, and several smaller service providers because they had ceased to exist. A third sampling and eligibility evaluation from a private sector provider (PSP) in Helsinki (*n* = 196) was added because roughly 7% of the 2nd phase sample based on LHR data proved to be initially diagnosed in the private sector (not included in NHR), mainly in the Helsinki area.

**Figure 1:**
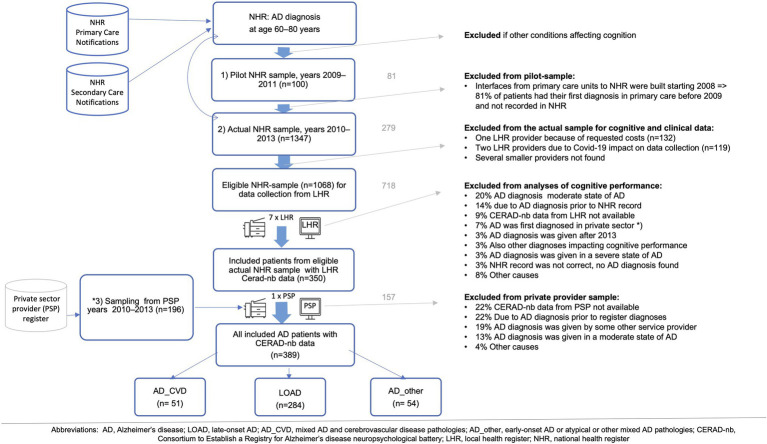
Sampling of patients with Alzheimer’s disease (AD) diagnosis.

After an eligible sample was identified, detailed diagnosis and cognitive screening data were requested from the LHRs and required for inclusion in the cognitive performance evaluation CERAD-nb. Figure 1 summarizes the sampling and exclusion criteria.

The final AD sample (aged 60–81 years) at the mild stage of AD with cognitive performance evaluations with CERAD-nb available comprised 389 patients diagnosed during years 2010–2013. AD was diagnosed according to the Finnish Current Care Guidelines for Memory Diseases (Working group set up by the Finnish Medical Society Duodecim 2016), which are based on the recommendations of NINCDS-ARDRA (McKhann et al., 1984) and DSM-IV (American Psychiatric Association 1994). The overall timespan for the data collection process including nationwide and local approvals, piloting, sampling, collecting, and saving of the data took 1 year 9 months from December 2018 until August 2020; out of which Covid-19 impacted timespan from March 2020 to May 2020.

The controls comprised persons aged 60–77 years having a Cardiovascular Risk Factors, Aging and Incidence of Dementia (CAIDE) Risk Score of 6 points or higher, indicating the presence of some modifiable risk factors, and who had participated in the FINGER study and had a screening visit during 2009–2011 (Ngandu et al., 2014). The exclusion criteria for the controls in this study were incomplete educational or CERAD-nb information (*n* = 59); conditions affecting cognitive performance at the screening visit or occurring during the first 5 years of the FINGER study (*n* = 171); and persons living in the area not included for the AD group (*n* = 360). After exclusions, 1980 patients served as controls. Control and AD samples included persons from both urban and rural areas as well as from southern, eastern, northern, middle, and western parts of Finland.

### Evaluating Eligibility of AD Patients

Seven LHR providers were visited in-person for evaluation of available clinical and cognitive data; one provider delivered the requested information on paper. For inclusion, mild stage of AD, Mini-Mental State Examination (MMSE), and the majority (max 2 missing) of CERAD-nb measures assessed less than 12 months prior to AD diagnosis were required. Component measures, such as separate word list trials or constructional copy/recall items or word list recognition yes/no items, were not obligatory. In case of more than one available CERAD-nb evaluation, the one nearest to the AD diagnosis was chosen.

Two primary care providers had CERAD-nb recorded in their LHRs in a structure that could have enabled electronic data transferring. All other providers had CERAD-nb scores written in variant text format either for all measures or for measures deviating from the cut-offs in electronic records or in separate paper archives.

### Classification of AD Stage and Subgroups

The methods for evaluating functional abilities were not uniform across hospital districts which reflects the difficulties in using clinically collected data for research purposes. The stage of AD was evaluated with the combination of MMSE (Folstein et al., 1975) and either the Global Deterioration Scale (GDS; Auer and Reisberg 1997) or the Clinical Dementia Rating Scale (CDR; Morris 1997) or written descriptions of independence in daily activities if no GDS nor CDR information was available. For the included mild AD patients with only MMSE available (*n* = 115), which was 29.5% out of all included mild AD patients (*n* = 389), a score of 20–30 was used to define mild AD, 10–19 moderate AD, and < 10 severe AD (Perneczky et al., 2006; Hänninen et al., 2010; Working group set up by the Finnish Medical Society Duodecim 2016). The AD subtypes and mixed dementia were identified from the diagnosis label written with or without the diagnosis code and inserted either in the specific entry fields or in the diagnosis description field of the system.

### Classification of Education

Educational attainment for the controls was reported both in years and with the highest degree. For the AD group educational information was available for 81% and occupation for 95% of patients. Education was categorized into three levels: former primary school including 7 years or less (lowest level); approximately 8–10 years of education, for example, studies in middle/technical/trade/vocational schools or matriculation examination without any other degree (middle level); and more than 10 years with a degree from college or university (highest level). For AD patients with only occupational information, the educational level was estimated based on occupation; the majority belonged to the lowest level, including occupations such as cleaner, bus driver, and stockman.

### CERAD-nb

CERAD-nb (Morris et al., 1989) is a relatively brief (i.e., 20–30 min) assessment tool including the following subtests: Verbal Fluency animal category, 15-item Boston Naming Test, MMSE, Word List Memory sum of three trials of 10 words each, Word List Recall and Recognition, Constructional Praxis Copy and Recall. The Finnish version of CERAD-nb also includes Clock Drawing (Hänninen et al., 1999, 2010; Pulliainen et al., 2007). Additional variables are calculated as follows: Word List Savings is the percentage of words retrieved on the delayed recall/the last learning trial; Word List Recognition is the sum of correct yes and no responses, and percentage of total 20; Verbal Memory total recall is the sum of word list delayed recall score and word list recognition score; and Constructional Praxis Savings is the Recall/Copy. Chandler et al. (2005) introduced a CERAD total score (Chandler total), which sums up Verbal Fluency (max 24), 15-item Boston Naming Test, Word List Memory, Recall and Recognition, and Constructional Praxis copy. Seo et al. (2010) further developed the Chandler total by adding the Constructional Praxis Recall (Seo total).

### Statistical Analyses

To analyze differences between nominal demographic variables between the AD group and the controls or between AD subgroups (Table 1), differences regarding gender, service provider, AD stage were analyzed by Pearson’s χ^2^ test. Ordinal age group differences between service providers or areas were analyzed by the Kruskal–Wallis test. Ordinal age group and education differences in Helsinki between Primary Care and Private service provider were analyzed by Mann–Whitney’s U-test.

**Table 1:** Study population included in cognitive performance evaluations.

		Total	Controls	AD total	LOAD	AD_CVD	AD_other
Total, *n* (%)		2369	1980	389	284	51	54
Gender^a^	Women, *n* (%)	1,247 (53)	1,022 (52)	225 (58)	166 (58)	28 (55)	31 (57)
	Men, *n* (%)	1,122 (47)	958 (48)	164 (42)	118 (42)	23 (45)	23 (43)
Education^b^	Missing value, *n* (%)	20 (1)		20 (5)	10 (4)	7 (14)	3 (6)
	Lowest level, *n* (%)	389 (16)	222 (11)	167 (43)	122 (43)	23 (45)	22 (41)
	Middle level, *n* (%)	1,152 (49)	1,027 (52)	125 (32)	94 (33)	15 (29)	16 (30)
	Highest level, *n* (%)	808 (34)	731 (37)	77 (20)	58 (20)	6 (12)	13 (24)
Age group^c^	60–69 years, *n* (%)	1,158 (49)	1,093 (55)	65 (17)	36 (13)	8 (16)	21 (39)
	70–74 years, *n* (%)	675 (28)	555 (28)	120 (31)	95 (33)	13 (25)	12 (22)
	75–81 years, *n* (%)	536 (23)	332 (17)	204 (52)	153 (54)	30 (59)	21 (39)
Cohort^d^	born 1930–1934, *n* (%)	364 (15)	224 (11)	140 (36)	107 (38)	23 (45)	10 (19)
	born 1935–1939, *n* (%)	683 (29)	535 (27)	148 (38)	111 (39)	17 (33)	20 (37)
	born 1940–1944, *n* (%)	698 (29)	630 (32)	68 (17)	51 (18)	6 (12)	11 (20)
	born 1945–1949, *n* (%)	618 (26)	591 (30)	27 (7)	15 (5)	4 (8)	8 (15)
	born 1950–1953, *n* (%)	6 (0)		6 (2)		1 (2)	5 (9)
Area^e^	Helsinki, *n* (%; AD group: primary care + private provider)	474 (20)	337 (17)	182 (47)	137 (48)	20 (39)	25 (46)
	Turku, *n* (%; AD group: primary care)	687 (29)	614 (31)	78 (20)	63 (22)	6 (12)	9 (17)
	Kuopio, *n* (%; AD group: primary + secondary care)	616 (26)	535 (27)	78 (21)	58 (21)	15 (29)	5 (9)
	Seinäjoki, *n* (%; AD group: secondary care)	355 (14)	277 (14)	34 (9)	20 (7)	8 (16)	6 (11)
	Oulu, *n* (%; AD group: primary + secondary care)	237 (10)	218 (11)	17 (3)	6 (2)	2 (4)	9 (17)
AD diagnosis given by^f^	Neurologist, *n* (%)			160 (41)	121 (43)	14 (27)	25 (46)
	Geriatrician, *n* (%)			136 (35)	103 (36)	21 (41)	12 (22)
	Physician of internal medicine, *n* (%)			53 (14)	42 (15)	4 (8)	7 (13)
	Other physician^*^, *n* (%)			40 (10)	17 (6)	12 (24)	10 (19)

*Controls, the non-demented comparison group; AD, Alzheimer’s disease; LOAD, late-onset AD; AD_CVD, mixed AD and cerebrovascular disease pathologies; AD_other, early-onset AD or atypical or other mixed AD pathologies; AD subgroup: LOAD, AD_CVD, AD_other. Lowest level: ≤7 years (former primary school); Middle level: ~8–10 years (e.g., middle/technical/trade/vocational school) or matriculation examination without any other degree; and Highest level: >10 years with degree from college or university*.

a *Gender: no difference (*p* = 0.153) between Controls and AD subgroups or between AD subgroups (*p* = 0.892)*.

b *Education: controls more educated than others (*p* < 0.001), no difference between AD subgoups (*p* = 0.068)*.

c *Age groups: controls younger than all others (*p* < 0.001), for AD subgroups AD_other younger than LOAD and AD_CVD (*p* < 0.01)*.

d *Cohorts differed between controls and AD subgroups (*p* < 0.001), additionally AD_other differed (*p* < 0.001) from LOAD and AD_CVD*.

e *Areas differed between controls and AD subgroups (*p* < 0.001). AD subgroups differed (*p* < 0.001); Kuopio had a larger AD_CVD group than statistically expected, Oulu had a larger AD_other group than statistically expected. Both areas included also secondary care units in addition to primary care*.

f *Diagnosis given by different specialists for AD subgroups (*p* < 0.05)*.

* *Other physicians included physicians for home care and primary care*.

For numeric CERAD-nb measures differences in cognitive performance between the AD group and the controls or between AD subgroups (Table 2), were evaluated by analyses of variance (ANOVA) and pairwise comparisons with Tukey correction. A value of *p* <0.05 was considered statistically significant, and for ANOVA corrected *p*-values were used. Values are given as means ± standard deviations (SD). All statistical analyses were performed using SPSS 26.0 (IBM Corp., Released 2019).

**Table 2:** Cognitive performance [mean, (SD)] for measures of the CERAD neuropsychological battery in the controls, AD group total, and AD subgroups.

CERAD-neuropsychological measures	controls *n* = 1980		25% of controls *n* = 495	10% of controls *n* = 198	5% of controls *n* = 99	2.5% of controls *n* = 50	1.5% of controls *n* = 30	AD group *n* = 389		LOAD *n* = 284		AD_CVD *n* = 51		AD_other *n* = 54		AD subgroup differences^*^
Verbal Fluency, animal category	23.1	(6.0)	19	16	15	12	11	13.9	(4.8)	14.3	(4.6)	12.5	(4.3)	13.1	(5.6)	
15-item Boston Naming Test	13.3	(1.7)	12	11	10	9	9	10.6	(2.9)	10.6	(2.8)	10.6	(2.8)	10.5	(3.1)	
MMSE	27.6	(1.9)	27	25	25	23	22	23.8	(2.4)	23.9	(2.4)	23.5	(2.6)	23.8	(2.4)	
Word List Memory	20.7	(3.6)	19	16	15	13	12	13.0	(3.6)	13.0	(3.5)	13.1	(3.2)	13.3	(4.5)	
Constructional Praxis Copy	9.9	(1.4)	9	8	7	7	6	9.2	(1.9)	9.4	(1.7)	8.5	(2.3)	9.4	(1.8)	^+^(*p* < 0.001). ^++^(*p* < 0.001)
Word List Recall (5 min)	6.8	(1.9)	6	4	4	3	2	2.4	(1.9)	2.3	(1.8)	2.6	(1.8)	3.1	(2.1)	^##^(*p* < 0.01)
Word List Savings (%)	83.7	(16.7)	75	63	57	43	33	44.7	(30.7)	42.0	(30.3)	47.9	(29.5)	56.6	(31.2)	^##^(*p* < 0.001)
Word List Recognition	19.1	(1.2)	19	17	17	16	15	16.0	(2.6)	15.9	(2.6)	16.5	(2.2)	16.6	(2.8)	^#^(*p* < 0.05). ^##^(*p* < 0.01)
Word List Recognition, %	95.6	(6.1)	95	85	85	80	75	80.2	(12.8)	79.3	(12.8)	82.6	(10.8)	83.1	(13.8)	^#^(*p* < 0.05). ^##^(*p* < 0.01)
Word List Recognition, yes	9.3	(1.1)	9	8	7	6	6	7.9	(2.1)	7.9	(2.1)	7.6	(2.0)	8.2	(1.9)	
Word List Recognition, no	9.8	(0.5)	10	9	9	8	8	8.3	(2.2)	8.0	(2.4)	9.0	(1.8)	9.3	(1.2)	^#^(*p* < 0.001). ^##^(*p* < 0.001)
Verbal Memory total recall	25.9	(2.7)	24	22	21	21	21	21.3	(3.3)	21.1	(3.3)	21.7	(2.7)	21.9	(3.8)	
Constructional Praxis Recall	8.9	(2.1)	7	6	6	4	4	4.7	(3.3)	4.6	(3.3)	4.2	(3.3)	5.7	(3.2)	^++^(*p* < 0.01). ^##^(*p* < 0.01)
Constructional Praxis Savings (%)	88.6	(16.1)	80	64	60	46	40	49.5	(32.9)	47.7	(32.3)	48.2	(35.5)	60.4	(32.6)	^++^(*p* < 0.05). ^##^(*p* < 0.001)
Clock Drawing	5.1	(1.3)	4	3	3	2	1	4.2	(1.6)	4.4	(1.5)	3.4	(1.9)	4.2	(1.6)	^+^(*p* < 0.001). ^++^(*p* < 0.05)
Chandler Total	80.3	(9.4)	74	68	63	59	57	55.7	(11.0)	56.1	(10.8)	52.4	(10.3)	56.8	(12.3)	
Seo Total	89.3	(10.4)	82	75	70	66	63	60.6	(12.7)	60.9	(12.6)	56.7	(12.0)	62.7	(13.8)	

*Controls, the non-demented comparison group; AD, Alzheimer’s disease; LOAD, late onset of AD; AD_CVD, mixed AD and cerebrovascular disease pathologies; AD_other, early onset of AD or atypical or other mixed AD pathologies. Symbols: + = AD_CVD < LOAD, ++ = AD_CVD < AD_other, # = LOAD < AD_CVD, ## = LOAD < AD_other. Chandler Total = Verbal Fluency + Boston Naming Test + Word List Memory + Constructual Praxis Copy + Word List Recall + Word List Recognition YES(−mistakes in YES). Seo Total = Chandler Total + Constructional Praxis Recall*.

* *Differences in cognitive performance were evaluated by analyses of variance (ANOVA) and pairwise post-hoc comparisons with Tukey correction*.

## Results

### Population

Altogether 1,072 NHR- and 196 PSP-based patients (total *n* = 1,268) with AD diagnosis were selected for the LHR search to collect information on AD diagnosis and cognitive function with CERAD-nb data. Of these, 70% originated from primary care, 15% from secondary care, and 15% from PSP.

The AD diagnosis was given at a moderate stage of AD for 19% of the NHR-based sample (*n* = 207, MMSE: mean 16.6 ± 2.6) and for 13% of the PSP sample (*n* = 25, MMSE: mean 17.1 ± 3.0). The primary care service providers differed regionally (*p* < 0.001); the proportion of AD patients at a moderate stage was largest in Turku (27.9% of the Turku sample) and Kuopio (20.2% of the Kuopio sample). Severe stage of AD (n = 30, MMSE: mean 7.9 ± 1.4) was found for 3% of the AD diagnoses in the eligible NHR-based sample and none in the PSP sample. There were more patients at a severe stage in Helsinki (4.5% of the Helsinki sample) than in the primary care providers in other regions (*p* < 0.011). CERAD-nb data from LHR were not available for altogether 11% of AD patients. Secondary care units used more comprehensive neuropsychological assessment for outpatients and MMSE for inpatients; the PSP register did not include any cognitive assessment information for these cases.

CERAD-nb data were found for 389 AD patients (31%) at a mild stage. Of the final 389 AD patients included, 78% originated from primary care, 12% from secondary care, and 10% from PSP. Women comprised 58% of the AD group and 52% of the controls. The mean age for the AD group was 74.0 ± 4.7 years and for the controls 68.5 ± 4.8 years. The NHR-based AD samples were similar in gender (*p* = 0.694) but differed in education (*p* < 0.001) and age (*p* < 0.001) between locations. Helsinki and Turku (=larger cities) had more educated patients than other locations (*p* < 0.01). Kuopio and Turku had the oldest patients and Helsinki and Oulu the youngest. In Helsinki, NHR and PSP AD samples were similar in gender (*p* = 0.137), but PSP patients were more educated (*p* < 0.014) and older (*p* < 0.001) than all NHR-based primary care patients. Information for one CERAD-nb screening was available for 68% of the included patients, while 32% had several CERAD-nb during different years/months.

For AD subgroups, diagnosis of LOAD was identified in 73% of the patients, AD_CVD in 13%, and the final 14% (combined as AD_other subgroup) consisted of 16 EOAD, 10 mixed Lewy body and AD pathologies, 2 AD patients with frontotemporal dementia, and 26 patients with AD of an unspecified mixed version. Table 1 presents the demographic data.

### Cognitive Performance

Table 2 presents the cognitive performance of the controls as means and as percentiles (25, 10, 5, 2.5, and 1.5) and of the different AD groupings as means. AD group performance was roughly at the 2.5–5th percentile of the controls for most verbal memory measures, around the 10th percentile for the 15-item Boston naming test and Word List Recognition yes. For Constructional Praxis Copy and Clock Drawing, the mean was roughly at the 25–50% level. The mean performance of the AD group was poorest for the summary measures of Chandler and Seo Total, which were at the 1.5th percentile. Within the AD group, cognitive performance differed between the service providers; the PSP group performed better than all other groups on MMSE (*p* = 0.026), Constructional Praxis Copy (*p* = 0.014), Constructional Praxis Recall (*p* = 0.014), Constructional Praxis Savings (*p* = 0.000), and Seo Total (*p* = 0.016).

The AD_CVD subgroup performed worse than the other AD subgroups on the Constructional Praxis Copy tasks and on Clock Drawing. The LOAD subgroup performed worse than the other subgroups on the Word List Recognition tasks. In addition, the AD_other subgroup performed better than the other subgroups on Constructional Praxis Savings and better than the LOAD subgroup on Word List Savings. The controls performed better than all AD subgroups on all subtasks other than Constructional Praxis Copy, on which the controls were better than AD_CVD and LOAD but did not differ from AD_other. Table 2 provides detailed descriptive statistics for all measured cognitive tasks by controls and AD subgroups.

## Discussion

We investigated cognitive screening data for 1,268 patients (aged 60–81 years) at the time of AD diagnosis during 2010–2013. Patients were identified from Finnish national health registers, and the sample was enlarged with a smaller private sector sample. Of the original sample, 31% were at a mild AD stage, with the CERAD-nb assessed and data documented. Local health records revealed also that a large proportion of AD patients (21%) receive AD diagnosis at a moderate or severe stage of the disease. Compared with controls, the largest difference on CERAD-nb was for total scores. Compared with the other AD subgroups, LOAD performed worst in Word List Recognition and AD_CVD in Constructional Praxis Copy and Clock Drawing. Real-world data combining Finnish national register data with clinical records can be used as a source to identify persons also for studies including cognitive data, although today the process is laborious.

A timely diagnosis is important for management and care of dementia not only from medication perspectives or long-term care cost perspectives but also enabling patients with AD and their families to engage with the diagnostic process at a time when they can most benefit from the information, advice and support given (Brooker et al., 2014; Gauthier et al., 2021). In the study that evaluated family caregivers’ experiences of timely AD diagnosis (Woods et al., 2019) of Finnish cases, 39% were classified as “diagnosis made when dementia was in the middle or late stage” and 49% of the caregivers reported that “it would have been better had the diagnosis been made earlier.” We found that the diagnosis was made at a moderate or severe stage of AD for 21% of our sample. The moderate stage at the time of diagnosis was overrepresented in those primary care units that had the oldest population. The severe stage at the time of diagnosis was overrepresented in Helsinki primary care, which might partly be because Helsinki primary care also includes large hospital for chronic care or because of the wider inclusion of patients from Helsinki (47%) to our sample. We also found that AD stage and measures of independence in daily activities were recorded to different health care system in very heterogeneous ways; harmonization is needed as it is a key component for measuring timely diagnosis. Additionally, for 32% of our included patients the CERAD-nb had been assessed (T3.2) several times. This may be appropriate in case of mild cognitive impairment and follow-up of conditions. However, it might have created at least for some patients delays compared with reported (Podhorna et al., 2020) mean time of diagnosis (T3.3) given within 6 months of presenting with symptoms (T3.1). Some part of the later recurrence might be due to out-of-date norms, cut-offs not sensitive enough for detecting cognitive difficulties in, e.g., highly educated persons, and differences in cognitive profiles between AD subgroups. Currently, the CERAD-nb cut-off scores with prioritized measures (Hänninen et al., 2010) are focused more on verbal measures, which identify LOAD but less well AD_CVD (Eckerström et al., 2020) or AD_other (Migliaccio et al., 2012; Palasí et al., 2015) subgroups. Further studies of CERAD-nb performance differences between AD subgroups are needed, also including evaluation of the necessity for adding other measures, like Word fluency F-A-S test and the Trail making A test (Eckerström et al., 2020), to the Finnish cognitive screening battery.

When comparing mean-level performance of the AD patients with that of controls, cognitive performance was at the level of 2.5–10% of the controls for most measures. The greatest differences were for Chandler and Seo total scores, for which mean-level performance of the AD group reached only 1.5% level of the controls. When comparing different subtypes of AD, we found that the LOAD subgroup (73% of patients) performed worse than others on Word List Recognition, and the AD_CVD-subgroup (13%) performed worse on Constructional Praxis Copy and Clock Drawing. As AD_other (14%) performed best also on Constructional Praxis Savings and Word List Savings, our findings support previous results (Migliaccio et al., 2012; Palasí et al., 2015) that progressive memory deficit occurs later for this group. When comparing service provider categories of the AD group, patients diagnosed in private sector performed better for several measures, as for MMSE; these patients were older but more educated than patients at other service providers. The easy access to the private sector may promote early detection of the disease but this may also reflect the better economic possibilities to use private sector services at the highest educational group.

The largest European register-based studies report mean age at the time of AD diagnosis to be 80–83 years (Tolppanen et al., 2016; Zakarias et al., 2019; Ford et al., 2020; Ponjoan et al., 2020). Based on age-specific dementia incidence rates (Prince et al., 2015) and Finnish population predictions for 2029 [Official Statistics of Finland (OSF) 2019], the majority (71%) of new AD diagnoses will occur among those aged 70–89 years, followed by those aged >90 years (21%); the phenomenon is expected to be even more pronounced during the 2030s. Therefore, it is essential to update/improve CERAD-nb with cognitive screening norms and cut-offs especially for the older age groups (Alenius et al., 2019). These also may explain, why during our data gathering we had challenges to cover control-based targets for age group 60–69 years, partly also 70–74 years but data for age-group 75–81 was easily available throughout all providers.

From a combined register and clinical data sampling perspective, our answer is “Yes” with some reservations to the question posed by Ponjoan and colleagues in their paper entitled “Is it time to use real-word data from primary care in Alzheimer’s disease?” (Ponjoan et al., 2020). It is possible to collect real-world data on AD using primary care registers, but based on our findings, enlarging primary care data with private sector data, and covering wider age range may lead to more representative data. Gathering screening data based on NHR criteria and combining LHR data in structured electronic format would speed up the needed processes. In addition, a relatively high proportion of patients had moderate/severe stage of the disease at the time of diagnosis, which is important to keep in mind when using register-based AD incidence as an outcome in research. Harmonization of screening procedures for independence in daily activities is also needed to secure timely diagnosis. More fine-tuned cognitive screening cut-offs may enhance timely diagnosis in the future; using outdated or unsensitive screening cut-offs would result in delay of the AD diagnosis. Age and education influence cognitive performance and they need to be taken into account when interpreting findings. Based on the data found in this study for the mild AD group and the controls, we have separately analyzed the effects of education, age, and gender on the CERAD-nb performance and created a basis for renewal of Finnish CERAD-nb cut-off scores (Alenius et al., 2022). Cultural barriers can be overcome with culturally appropriate, translated, and validated cognitive assessment tools (Gauthier et al., 2021); a good example of this is the tool developed by the10/66 study group (Prince et al., 2003; Prina et al., 2019).

### Strengths and Limitations

The main strength of our study is the wide-ranging sampling of patients, covering the national, local, and private sector providers and both urban and rural areas. Our real-world AD population consisted of all kinds of clinical patients compared with specialized clinic-based studies or research cohorts, which may be less representative and focus on certain diagnostic groups or educational or health conditions.

This study has several limitations. First, AD was diagnosed during years 2010–2013 according to the Finnish Current Care Guidelines for Memory Diseases criteria based on older recommendations; after these criteria for AD diagnosis have been extensively revised. Second, as no measures nor written text related to GDS/CDR/written capability for daily activities was available for 29% of the included AD sample, also patients with a moderate stage of AD but MMSE ≥ 20 might have been included for cognitive performance evaluation. Thus, the results describing cognitive performance among patients with AD may be slightly lower than they would have been if only patients in mild stage were included. Furthermore, our study was planned to collect a sufficient sample size for each education, age, and gender group, but this was not achieved for the age group 60–69 years. In addition, the current data collection did not include those aged over 80 years, limiting the generalizability of the findings. In addition, the AD_other group was relatively small and a combination of many different AD and mixed variants, which impacted the cognitive profile of that group. Finally, the technical challenges of primary care interfaces impacting the AD diagnoses given before the year 2010 may have hampered the comparability within different service providers of AD samples.

## Conclusion

Of the original sample (*n* = 1,268), 31% were at a mild AD stage, with CERAD-nb assessed and data documented. Local health records revealed also that 21% received an AD diagnosis at a moderate or severe stage of the disease. In the AD subgroups, LOAD patients performed worst in Word List Recognition and AD_CVD patients in Constructional Praxis Copy and Clock Drawing. A combination of national and local health records can be used to collect data relevant for cognitive screening. Currently, the process is laborious, but it could be improved with refined search algorithms and electronic LHR data. On a nationwide scale, further CERAD-nb studies are needed to create and update norms and cut-offs for dementia screening for new and better-educated generations of elderly persons and for the expanding age groups 80–89 years and ≥90 years.

## Data Availability Statement

The datasets presented in this article are not readily available because data can be made available only for those fulfilling the requirements for viewing confidential data as required by Finnish law and the Finnish Institute for Health and Welfare. Moreover, the purpose of the research must be in alignment with the informed consent provided for this study and/or the FINGER study (controls), with Finnish law and regulations at the Finnish Institute for Health and Welfare. Requests are to be submitted to the Finnish Institute for Health and Welfare: kirjaamo@thl.fi. Requests to access the datasets should be directed to kirjaamo@thl.fi.

## Ethics Statement

The studies involving human participants were reviewed and approved by the Finnish Institute for Health and Welfare (THL), decision THL/1649/6.02.00/2018. As a register-based study, no informed consent was separately obtained. For the controls, a statement of ethics concerning the FINGER participants (with Clinical Trials identifier: NCT01041989) has been provided elsewhere, see article “The Finnish geriatric intervention study to prevent cognitive impairment and disability (FINGER): Study design and progress.” Written informed consent for participation was not required for this study in accordance with the national legislation and the institutional requirements.

## Author Contributions

TN and MA prepared the study design and sampling. MA took care of the data acquisition, data management, and statistical analyses. TN, LH, SK, and MA prepared the conceptualization for key content. MA wrote the manuscript drafts for review and comments by TN, LH, and SK. Other authors reviewed the text and gave their input to the interpretation of the results. TN, TH, and MKi are members of the FINGER study. Additionally, TH, LH, and MKa participated in the pioneer studies of Finnish cognitive screening. All authors contributed to the article and approved the submitted version.

## Funding

Minna Alenius was supported during 2020 by the Finnish Brain Foundation and during 2021 by the Finnish Alzheimer’s Disease Research Society. FINGER data collection was funded by the Academy of Finland, the Finnish Social Insurance Institution, the Juho Vainio Foundation, the Yrjö Jahnsson Foundation, and the Alzheimer’s Research and Prevention Foundation.

## Conflict of Interest

The authors declare that the research was conducted in the absence of any commercial or financial relationships that could be construed as a potential conflict of interest.

## Publisher’s Note

All claims expressed in this article are solely those of the authors and do not necessarily represent those of their affiliated organizations, or those of the publisher, the editors and the reviewers. Any product that may be evaluated in this article, or claim that may be made by its manufacturer, is not guaranteed or endorsed by the publisher.
